# Diagnosis of Thoracic Aortic Aneurysms by Computed Tomography Without Allometric Scaling

**DOI:** 10.1001/jamanetworkopen.2020.23689

**Published:** 2020-11-03

**Authors:** Sameh Yousef, Makoto Mori, Syed Usman Bin Mahmood, Roland Assi, Prashanth Vallabhajosyula, Arnar Geirsson, George Tellides

**Affiliations:** 1 Department of Surgery, Yale University School of Medicine, New Haven, Connecticut

## Abstract

This diagnostic study of computed tomography scans at a single tertiary care center compares radiologist descriptions of abnormal size ascending aortas with definitions of aorta diameters defined by nomograms.

## Introduction

Thoracic aortic aneurysms (TAA) are defined as aorta diameter greater than 1.5 times the expected size by 11 stakeholder medical societies.^1,2^ Enlarged aortas less than 1.5 times the expected size are termed *ectasia*,^1,2^ whereas aortas within 2 standard deviations of population means are considered normal.^3,4^ Dilatation is synonymous with ectasia or aneurysm.^5^ A logistical challenge in diagnosing TAA according to current definitions is the dependence of expected aorta diameter on age, sex, and body size, thus requiring nomograms.^1,2,3,4^ Accurate diagnosis of TAA is important because overdiagnosis (eg, diagnosing aneurysm in cases of ectasia) or misdiagnosis (eg, diagnosing aneurysm or ectasia when normal) may cause unnecessary patient anxiety, unwarranted tests, and excessive referrals. Motivated by inappropriate consultations, we hypothesized that aortas diagnosed as abnormal by computed tomography (CT) are not routinely normalized for patient characteristics, and we evaluated whether radiological diagnoses are concordant with nomogram-based definitions of TAA.

## Methods

In this diagnostic study, we retrospectively reviewed chest CT scans of patients aged 50 to 85 years obtained for any indication at a single tertiary care center, Yale New Haven Hospital, during a 4-year period from February 1, 2013, to December 30, 2016. Radiology reports were screened for descriptors of abnormal size, identified records were reviewed, and ascending aortas described as increased in size were remeasured at maximal outer-to-outer diameter by double-oblique method. Expected aortic diameters were derived from published nomograms.^3^ Data were analyzed in July and August 2019. *P* < .05 was considered statistically significant in 2-sided tests. The study was approved by Yale’s Human Investigation Committee with a waiver for informed consent because of the minimal risk nature of the study. This study follows the Standards for Reporting of Diagnostic Accuracy (STARD) reporting guideline.

## Results

**Figure 1.**
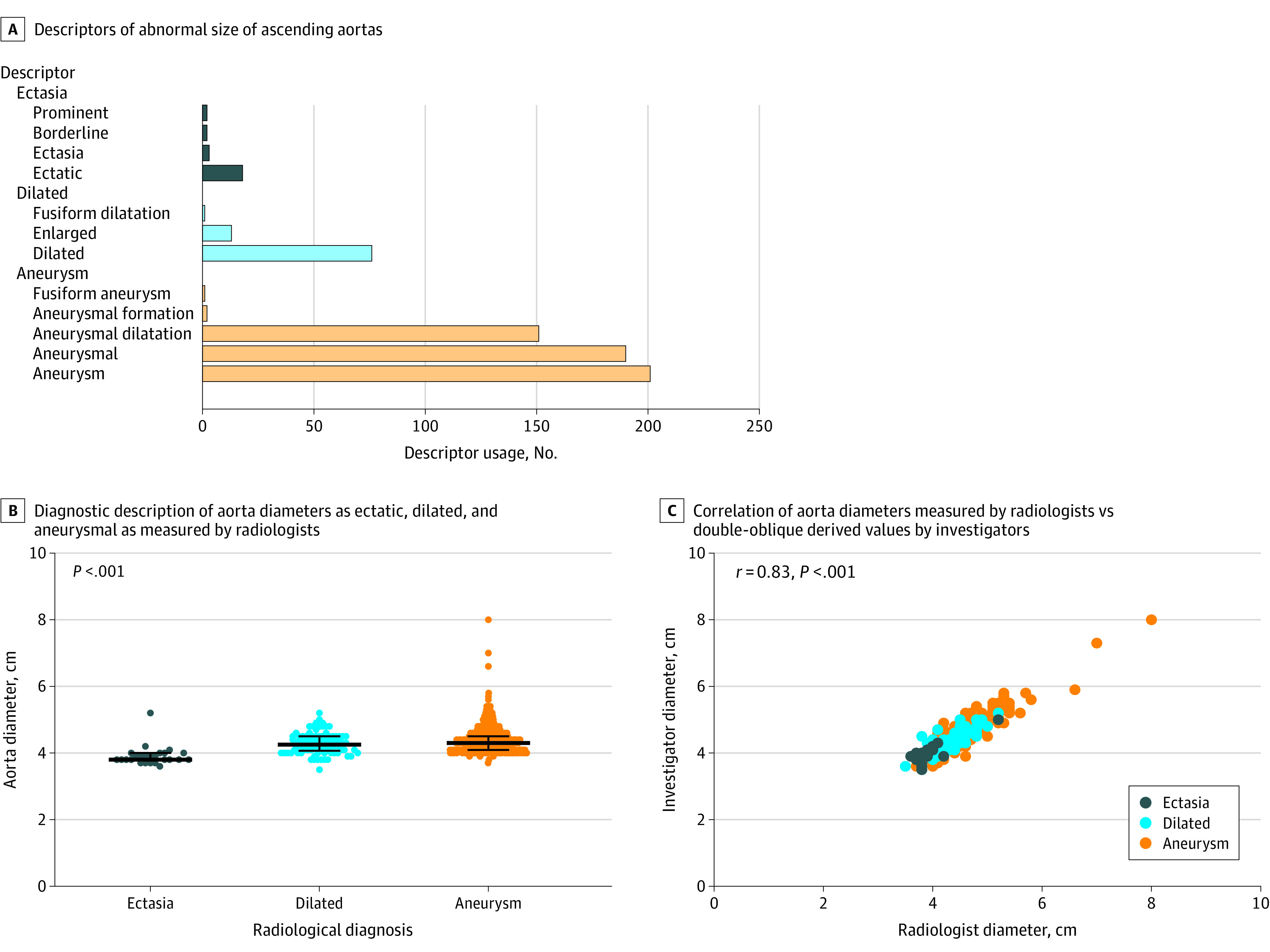
Radiological Diagnosis of Thoracic Aortic Aneurysms B, Individual data are shown with overlying bars representing medians with interquartile ranges. Data are not normally distributed by Shapiro-Wilk test; *P* < .05 is considered significant by Kruskal-Wallis test with Dunn multiple comparison test. C, *P* <.05 is considered significant by Spearman correlation.

**Figure 2.**
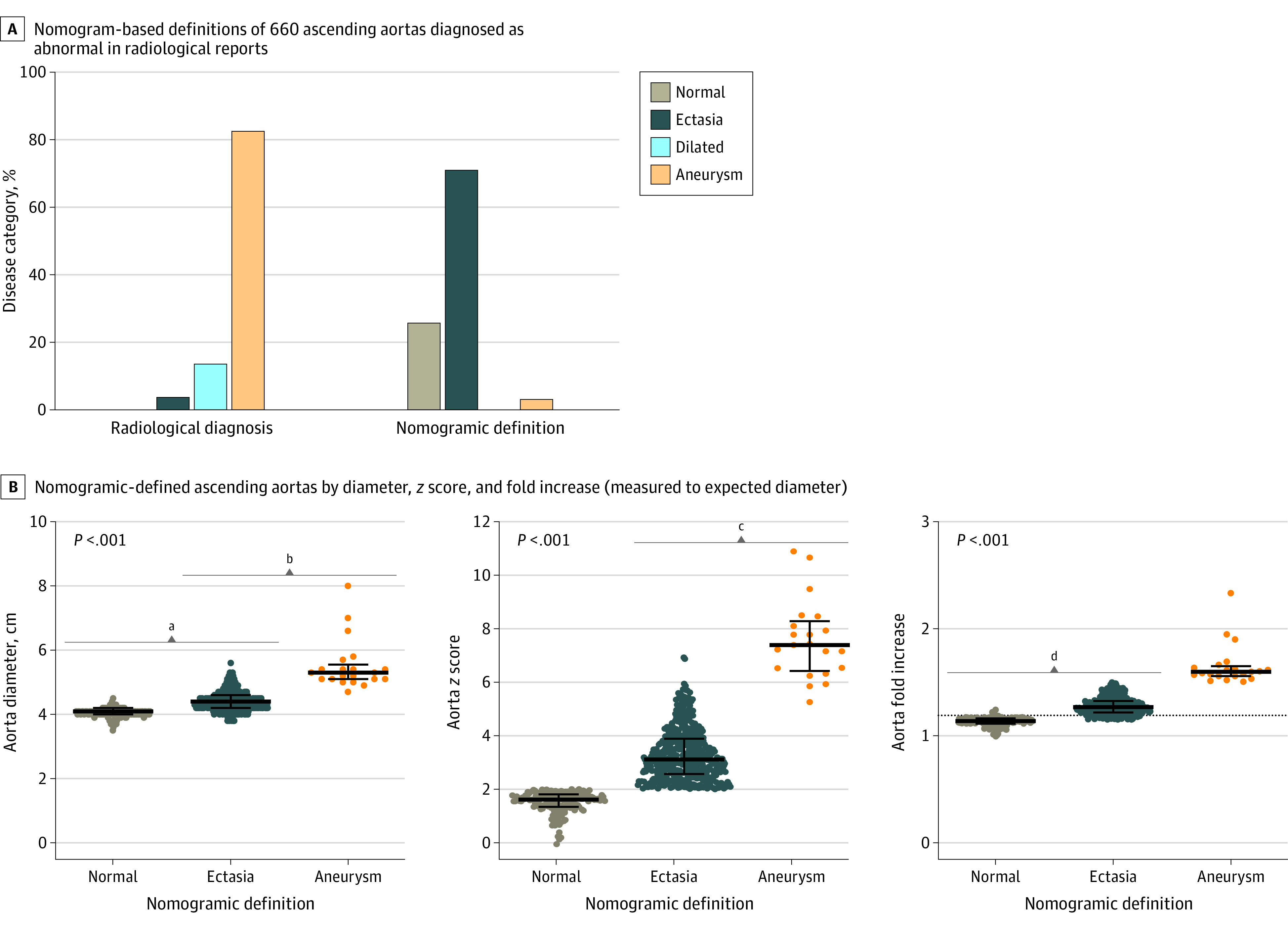
Nomogramic Definitions of Thoracic Aortic Aneurysms B, Individual data are shown with overlying bars representing medians with interquartile ranges. The dotted line indicates 1.2-fold increase in diameter. Data are not normally distributed by Shapiro-Wilk test; *P* < .05 is considered significant by Kruskal-Wallis test with Dunn multiple comparison test, and overlap coefficient (∩) by Szymkiewicz-Simpson test (overlap coefficients calculated as the size of the intersection divided by the smaller of the size of 2 sets, where 0 is no overlap and 1 is complete overlap; coefficients = 0 are not shown). ^a^∩ =  0.96. ^b^∩ = 0.76. ^c^∩ = 0.33. ^d^∩ = 0.36.

Of 21 320 CT scans in unique patients (mean [SD] age, 70.4 [8.9] years; 16 203 [76%] male; and mean [SD] body surface area, 1.96 [0.27] m^2^), 660 radiology reports (3.1%) described an abnormal size ascending aorta (9 reports with incomplete data and 66 reports of abnormal segments other than the ascending aorta were not included). Twelve descriptors were recorded (Figure 1); the most frequent was aneurysm, while dilatation and ectasia were less common. Aortas described as aneurysmal or dilated were larger than ectatic aortas but with substantial overlap of diameters. There was a correlation between aorta diameters recorded in radiology reports with confirmatory investigator measurements, with a mean (SD) difference of 0.03 (0.18) cm (*r* = 0.83). Radiological diagnoses, however, differed markedly from nomogramic definitions (Figure 2). Among 545 aortas with aneurysm descriptors, only 19 (3.5%) met a nomogram-based definition of TAA, while 131 (24.0%) were normal. Furthermore, of 90 aortas described as dilatated, 2 (2.2%) were aneurysmal and 23 (25.6%) were normal, whereas of 25 aortas described as ectatic, none were aneurysmal and 15 (60.0%) were normal. Transition from normal to ectasia occurred at diameters of 3.8 to 4.5 cm (*z* score, 2.0; a 1.15- to 1.24-fold increase) while transition from ectasia to aneurysm occurred at diameters of 4.7 to 5.6 cm (*z* score, 5.2-6.9; a 1.5-fold increase). A 1.2-fold increase in diameter differentiated ectatic from normal aortas with similar overlap as *z* score differentiated aneurysmal from ectatic aortas.

## Discussion

This diagnostic study found differences in the diagnosis of TAA when aorta diameter is the only indicator vs allometric scaling. Normal aortas represented 25% of abnormal radiological diagnoses, and nomogramic classification identified 25-fold fewer aortic aneurysms.

Variable terms are used to describe enlarged aortas with a predominance of advanced disease descriptors. To simplify nomogram-based diagnosis of TAA, we developed a calculator suitable for individual patients or large databases.^6^ We submit that an alternative criterion to define the lower limit of ectasia is greater than 1.2 times expected aortic diameter, which complements the accepted upper limit definition.

This study had several limitations, including its retrospective and single-center design, undocumented radiologist methods to measure aorta diameter, and use of the aorta calculator for diagnostic but not therapeutic decision-making. In conclusion, our findings suggest that ectasia should be consistently used to describe enlarged aortas that are not aneurysmal and normalized aortic diameters should be used in the diagnosis of TAA.
